# Implementation of the new easy approach to fuzzy multi-criteria decision aid in the field of management

**DOI:** 10.1016/j.mex.2021.101344

**Published:** 2021-04-13

**Authors:** Paweł Ziemba

**Affiliations:** Faculty of Economics, Finance and Management, University of Szczecin, Cukrowa 8, Szczecin 71-004, Poland

**Keywords:** Multi-criteria decision making, MATLAB, NEAT F-PROMETHEE, Uncertainty, Fuzzy sets, Outranking relation

## Abstract

Decision-making is one of the most important management functions and a critical task for managers. The tools that support decision makers in making decisions are Multi-criteria Decision Making/Aid/Analysis (MCDM/MCDA) methods. Since most decisions are made under conditions of uncertainty, the fuzzy MCDM/MCDA methods are particularly important as they allow capturing the uncertainty and imprecision of the information used in making decisions. This method is the Fuzzy Preference Ranking Organization Method for Enrichment Evaluation (Fuzzy PROMETHEE), and its extension in the form of New Easy Approach to Fuzzy PROMETHEE (NEAT F-PROMETHEE). However, the unavailability of software using the NEAT F-PROMETHEE method significantly reduces its ease of use and may discourage potential users and researchers considering using it in their studies. Therefore, to facilitate the use of this MCDA method, the article presents the implementation of NEAT F-PROMETHEE in the MATLAB environment. Moreover, the verification of the developed implementation and its application in the management decision-making problem is presented, together with the analysis of the operation of the mapping correction function used in NEAT F-PROMETHEE. The results obtained with NEAT F-PROMETHEE were compared with the results of the Fuzzy PROMETHEE method which did not apply correction. The analysis shows that the correction applied in NEAT F-PROMETHEE allows obtaining results with a smaller error than the non-corrected implementations of PROMETHEE Fuzzy. Therefore, a more accurate solution of the decision problem is obtained.

• improving the process of mapping fuzzy numbers in the Fuzzy PROMETHEE method
• implementing a correction mechanism while mapping trapezoidal fuzzy numbers

## Graphical abstract

**Figure:**
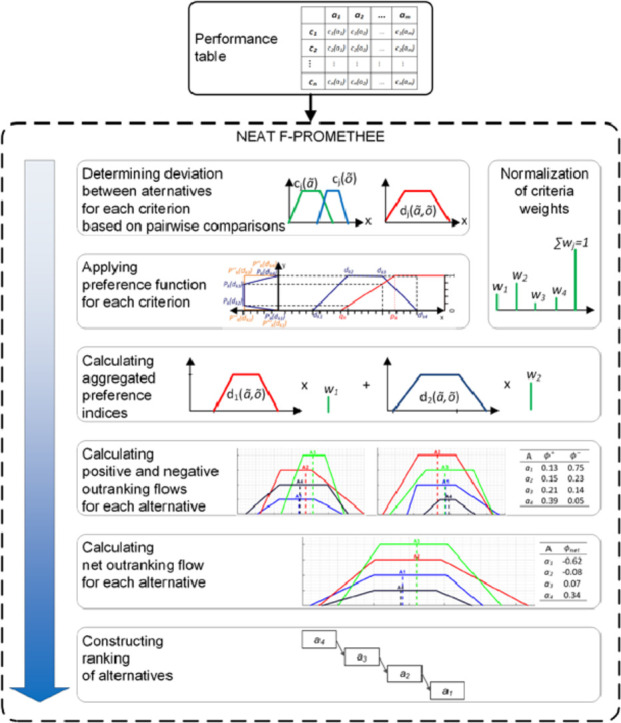

Specifications table

**Table:** 

Subject Area	Computer Science
More specific subject area	*Decision Support System in Management*
Method name	*PROMETHEE – Preference Ranking Organization METHod for Enrichment Evaluation*
Name and reference of original method	*Brans, J.P., & De Smet, Y. (2016). PROMETHEE Methods. In S. Greco, M. Ehrgott, & J.R. Figueira (Eds.), Multiple Criteria Decision Analysis. State of the Art Surveys (2nd ed.) (pp. 187–219). New York, Springer*[2] *Geldermann, J., Spengler, T., & Rentz, O. (2000). Fuzzy outranking for environmental assessment. Case study: iron and steel making industry. Fuzzy Sets and Systems, 115(1), 45–65.* doi:10.1016/S0165-0114(99)00021-4[3]

## Method details

Decision-making is inseparable from management [4], and some researchers even claim that it is the most important function of management [5] and the most important task of managers [6]. In turn, most of the nontrivial management decision-making problems are of a multi-criteria nature, and Multi-criteria Decision Making/Aid/Analysis (MCDM/MCDA) methods in the fuzzy or crisp forms are used to solve them. Fuzzy methods, unlike crisp methods, allow to capturing uncertainty and imprecision [7], usually occurring in management decisions [6]. Therefore, fuzzy methods are widely used in management [8]. One of the MCDA methods often used in management problems [9] is the Preference Ranking Organization Method for Enrichment Evaluation (PROMETHEE) [2], and its fuzzy or stochastic developments [10,11]. This is due to the ease of use and universality of the PROMETHEE method, transparency of its calculation procedure and high usefulness of the fuzzy versions of PROMETHEE in decision-making problems characterized by uncertainty [10,^12^,13]. The method which is a fuzzy development of PROMETHEE is New Easy Approach to Fuzzy PROMETHEE (NEAT F-PROMETHEE). This method, based on trapezoidal fuzzy numbers, has similar transparency and ease of use as the sharp PROMETHEE method. Moreover, it meets the methodological assumptions of the original PROMETHEE method, and thus gives the possibility to apply preference functions according to crisp PROMETHEE, as well as retaining appropriate scales for preference degrees and outranking flows. NEAT F-PROMETHEE uses six different preference functions, allows the use of linguistic, crisp and fuzzy values, in their natural scales, and gives the possibility of obtaining partial and total order of alternatives, thus offering great versatility. Finally, by applying the correction in the preference functions, it reduces the approximation errors that arise in other fuzzy PROMETHEE implementations when mapping fuzzy deviation to the form of a unicriterion preference degree. As a result, the NEAT F-PROMETHEE method is widely used in solving management decision problems [1,^10^,^14^,15]. On the other hand, the unavailability of software using the NEAT F-PROMETHEE method significantly reduces its ease of use and discourages potential users and researchers considering using NEAT F-PROMETHEE in their studies. Therefore, an important practical issue is the implementation of the NEAT F-PROMETHEE method in the programming language commonly used by researchers.

This article presents theoretical basis, technical details and MATLAB implementation of the NEAT F-PROMETHEE method. In the following part of the article there is a description of calculation procedures used in the method together with relevant mathematical equations and codes implementing these procedures in the MATLAB environment. The article ends with both the validation of the method based on the application of the developed MATLAB implementation in order to solve the decision problem and the analysis of obtained results.

### Input data

The NEAT F-PROMETHEE method is a discrete MCDA method that addresses the problem of ranking *m* of fuzzy decision alternatives belonging to the set $\tilde{A}=\left\{\tilde{a_{1}},\tilde{a_{2}},\ldots ,\tilde{a_{m}}\right\}$ using n criteria, belonging to the set $C=\{c_{1},c_{2},\ldots ,c_{n}\}$. It is based on trapezoidal fuzzy numbers (TFNs) in the form of $\tilde{FN}=\left(FN_{1},FN_{2},FN_{3},FN_{4}\right)$, for which the membership function is described by the formula (1):

(1) $$\mu_{\tilde{FN}}\left(x\right)=\left\{ \begin{array}{c} \frac{x-FN_{1}}{FN_{2}-FN_{1}}\Leftrightarrow FN_{1}\leq x<FN_{2}\\ 1\;\Leftrightarrow FN_{2}\leq x\leq FN_{3}\;\\ \frac{x-FN_{4}}{a_{3}-FN_{4}}\Leftrightarrow FN_{3}<x\leq FN_{4}\\ 0\;\Leftrightarrow otherwise \end{array}\right.$$

Firstly, the fuzzy weights of the criteria are obtained $\tilde{Wf}=\left\{\tilde{wf_{1}},\tilde{wf_{2}},\ldots ,\tilde{wf_{n}}\right\}$ and the fuzzy values of the alternatives for each criterion. Both weights and values of alternatives can be expressed on natural scales of specific criteria (e.g. PLN or $ for Price), or on linguistic scales. The following is the content of the *LingVal.m* file, which defines the linguistic scales for weights of criteria and values of alternatives.



**Fig. 1:**
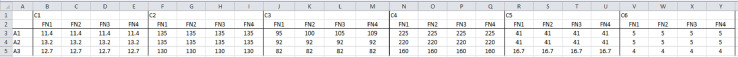
The structure of the alternatives.xlsx file containing the values of alternatives for individual criteria.

In turn, Fig. 1 shows the structure of the file *alternatives.xlsx*, from which the values of alternatives are loaded into the performance matrix.

The structure of the performance matrix *E* is described by the formula (2):

(2) $$E=\begin{array}{c} {\tilde{a}}_{1}\\ {\tilde{a}}_{2}\\ \vdots \\ {\tilde{a}}_{m} \end{array}\left[\begin{array}{cccc} c_{1} & c_{2} & \cdots & c_{n}\\ {\tilde{e}}_{1,1} & {\tilde{e}}_{1,2} & \cdots & {\tilde{e}}_{1,n}\\ {\tilde{e}}_{2,1} & {\tilde{e}}_{2,2} & \cdots & {\tilde{e}}_{2,n}\\ \vdots & \vdots & \ddots & \vdots \\ {\tilde{e}}_{m,1} & {\tilde{e}}_{m,2} & \cdots & {\tilde{e}}_{m,n} \end{array}\right]$$

where $\tilde{e_{i,j}}=c_{j}\left(\tilde{a_{i}}\right)$, $\tilde{e_{i,j}}=\left({e_{i,j}}_{1},{e_{i,j}}_{2},{e_{i,j}}_{3},{e_{i,j}}_{4}\right)=\left(c_{j}\left({a_{i}}_{1}\right),c_{j}\left({a_{i}}_{2}\right),c_{j}\left({a_{i}}_{3}\right),c_{j}\left({a_{i}}_{4}\right)\right)$, therefore $\tilde{e_{i,j}}$ represents the performance level of an alternative $\tilde{a_{i}}$ according to a criterion $c_{j}$.

In addition to the values of the alternatives and the weights of the criteria, preference directions are defined at the beginning (for the 'profit' criteria, the maximum is preferred and for the 'cost' criteria the minimum is preferred), as well as preference functions and thresholds related to the preference functions (indifference (*q*), preference (*p*), Gaussian (*s*)) for individual criteria are also defined. As a result, a complete model of the decision maker's preferences and, more broadly, a model of the decision-making problem is constructed. For the model developed in this way, in subsequent stages, calculations of the NEAT F-PROMETHEE method are performed, alternatives are ranked and results are displayed. The script code, including the indicated actions, is presented below.



### Calculations of NEAT F-PROMETHEE

Calculations for the NEAT F-PROMETHEE method are performed in several steps. First, the performance matrix *E* is transformed in such a way that the direction of preferences for each criterion is maximum. For this purpose, the values of the 'cost' criteria are transformed according to the formulae (3)(4):

(3) $$\tilde{e_{i,j}}=\tilde{e_{i,j}}\times \left(-1\right)=\left(-{e_{i,j}}_{1},-{e_{i,j}}_{2},-{e_{i,j}}_{3},-{e_{i,j}}_{4}\right)$$

(4) $$\tilde{e_{i,j}}=\left(-{e_{i,j}}_{4},-{e_{i,j}}_{3},-{e_{i,j}}_{2},-{e_{i,j}}_{1}\right)$$

In the subsequent steps, fuzzy deviations are calculated and mapped to the form of unicriterion preference degrees and the defuzzification and normalisation of weights, preference aggregation and calculation of fuzzy outranking flows and defuzzification of calculated outranking flows take place. The code of the main NEAT F-PROMETHEE function is presented below.



The calculation of fuzzy deviations is carried out for each pair of alternatives for each criterion. It shall be carried out according to the formula (5):

(5) $$\Lambda_{\tilde{a_{i}},\tilde{a_{j}}\in \tilde{A}}\Lambda_{c_{k}\in C}\tilde{d_{k}}\left(\tilde{a_{i}},\tilde{a_{j}}\right)=c_{k}\left(\tilde{a_{i}}\right)⊖c_{k}\left(\tilde{a_{j}}\right)=\tilde{e_{i,k}}⊖\tilde{e_{j,k}}\;=\left({e_{i,k}}_{1},{e_{i,k}}_{2},{e_{i,k}}_{3},{e_{i,k}}_{4}\right)⊖\left({e_{j,k}}_{1},{e_{j,k}}_{2},{e_{j,k}}_{3},{e_{j,k}}_{4}\right)=\left({e_{i,k}}_{1}-{e_{j,k}}_{4},{e_{i,k}}_{2}-{e_{j,k}}_{3},{e_{i,k}}_{3}-{e_{j,k}}_{2},{e_{i,k}}_{4}-{e_{j,k}}_{1}\right)$$

**Fig. 2:**
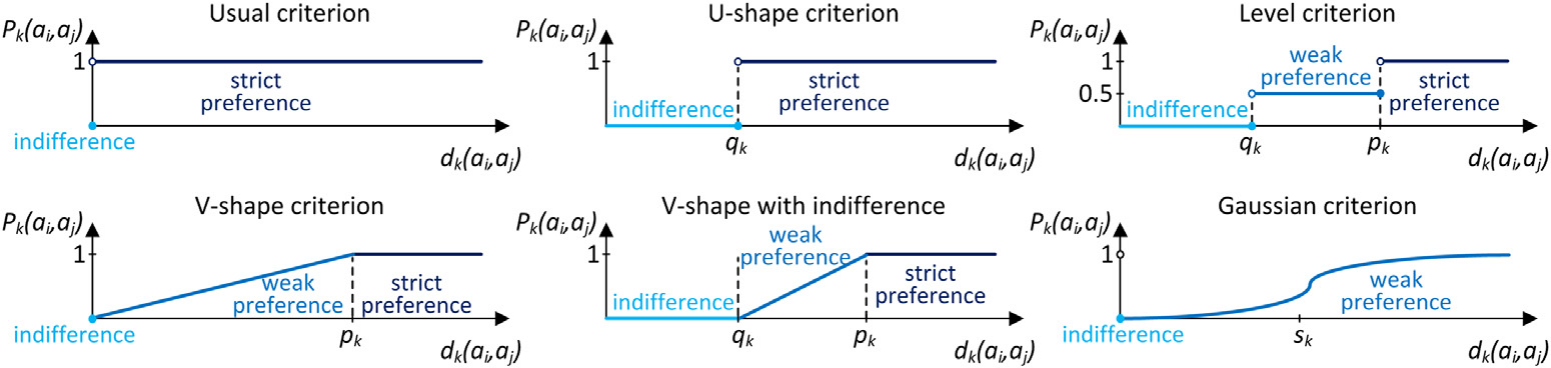
Preference functions of the PROMETHEE method.

Then, the fuzzy deviations obtained are mapped using the appropriate preference function. In the classic crisp PROMETHEE method, six preference functions are used, shown in Fig. 2.

**Fig. 3:**
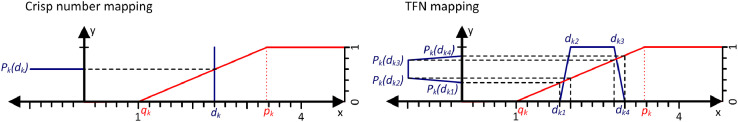
The mapping of a crisp number and a TFN.

The mapping of crisp numbers consists in calculating the value of the preference function $P_{k}$ for the deviation $d_{k}$, so the function $P_{k}\left(d_{k}\right)$ is calculated. For TFNs, four values of the deviation $\tilde{d_{k}}=\left({d_{k}}_{1},{d_{k}}_{2},{d_{k}}_{3},{d_{k}}_{4}\right)$ are already mapped and thus four values are obtained $\tilde{P_{k}}\left(\tilde{d_{k}}\right)=\left(P_{k}\left({d_{k}}_{1}\right),P_{k}\left({d_{k}}_{2}\right),P_{k}\left({d_{k}}_{3}\right),P_{k}\left({d_{k}}_{4}\right)\right)$. The comparison of the mapping of crisp numbers and TFNs is shown in Fig. 3.

In the case of crisp numbers, used in the classic PROMETHEE method, the preference functions allow precise mapping of the deviation value $d_{k}$ to the form of the unicriterion preference degree $P_{k}\left(d_{k}\right)$. But in the case of TFNs, used in many fuzzy versions of PROMETHEE, approximation errors can occur during mapping. Therefore, the NEAT F-PROMETHEE method extends the mapping process with a function to correct mapping errors. The preference functions together with the correction functions used in NEAT F-PROMETHEE are shown in the formulae 6–(17).

Usual criterion (6):

(6) $$\tilde{P_{k}}\left(\tilde{d_{k}}\right)=\left(P_{k}\left({d_{k}}_{1}\right),P_{k}\left({d_{k}}_{2}\right),P_{k}\left({d_{k}}_{3}\right),P_{k}\left({d_{k}}_{4}\right)\right)=\left\{ \begin{array}{c} 0\Leftrightarrow d_{k_{l}}\leq 0\;\\ 1\Leftrightarrow d_{k_{l}}>0\; \end{array},\right.\;l=1,\ldots ,4$$

Correction for usual criterion (7):

(7) $$\left\{ \begin{array}{c} P_{k}\left({d_{k}}_{2}\right)=0⇐\frac{-{d_{k}}_{1}}{{d_{k}}_{2}-{d_{k}}_{1}}>0.5\wedge {d_{k}}_{1}<0\leq {d_{k}}_{2}\\ P_{k}\left({d_{k}}_{3}\right)=1⇐\frac{-{d_{k}}_{4}}{{d_{k}}_{3}-{d_{k}}_{4}}>0.5\wedge {d_{k}}_{3}\leq 0<{d_{k}}_{4} \end{array}\right.$$

U-shaped criterion (8):

(8) $$\tilde{P_{k}}\left(\tilde{d_{k}}\right)=\left(P_{k}\left({d_{k}}_{1}\right),P_{k}\left({d_{k}}_{2}\right),P_{k}\left({d_{k}}_{3}\right),P_{k}\left({d_{k}}_{4}\right)\right)=\left\{ \begin{array}{c} 0\Leftrightarrow d_{k_{l}}\leq q_{k}\;\\ 1\Leftrightarrow d_{k_{l}}>q_{k}\; \end{array}\right.,\;l=1,\ldots ,4$$

Correction for U-shaped criterion (9):

(9) $$\left\{ \begin{array}{c} P_{k}\left({d_{k}}_{2}\right)=0⇐\frac{q_{k}-{d_{k}}_{1}}{{d_{k}}_{2}-{d_{k}}_{1}}>0.5\wedge {d_{k}}_{1}<q_{k}\leq {d_{k}}_{2}\\ P_{k}\left({d_{k}}_{3}\right)=1⇐\frac{q_{k}-{d_{k}}_{4}}{{d_{k}}_{3}-{d_{k}}_{4}}>0.5\wedge {d_{k}}_{3}\leq q_{k}<{d_{k}}_{4} \end{array}\right.$$

V-shaped criterion (10):

(10) $$\tilde{P_{k}}\left(\tilde{d_{k}}\right)=\left(P_{k}\left({d_{k}}_{1}\right),P_{k}\left({d_{k}}_{2}\right),P_{k}\left({d_{k}}_{3}\right),P_{k}\left({d_{k}}_{4}\right)\right)=\left\{ \begin{array}{c} 0\Leftrightarrow d_{k_{l}}\leq 0\\ \frac{d_{k_{l}}}{p_{k}}\Leftrightarrow 0<d_{k_{l}}\leq p_{k}\\ 1\Leftrightarrow d_{k_{l}}>p_{k}\; \end{array}\right.,\;l=1,\ldots ,4$$

Correction for V-shaped criterion (11):

(11) $$\left\{ \begin{array}{c} P_{k}\left({d_{k}}_{2}\right)=0⇐\frac{-{d_{k}}_{1}}{{d_{k}}_{2}-{d_{k}}_{1}}>0.5\wedge {d_{k}}_{1}<0\leq {d_{k}}_{2}\\ P_{k}\left({d_{k}}_{3}\right)=1⇐\frac{p_{k}-{d_{k}}_{4}}{{d_{k}}_{3}-{d_{k}}_{4}}>0.5\wedge {d_{k}}_{3}\leq p_{k}<{d_{k}}_{4} \end{array}\right.$$

Level criterion (12):

(12) $$\tilde{P_{k}}\left(\tilde{d_{k}}\right)=\left(P_{k}\left({d_{k}}_{1}\right),P_{k}\left({d_{k}}_{2}\right),P_{k}\left({d_{k}}_{3}\right),P_{k}\left({d_{k}}_{4}\right)\right)=\left\{ \begin{array}{c} 0\Leftrightarrow d_{k_{l}}\leq q_{k}\\ \frac{1}{2}\Leftrightarrow q_{k}<d_{k_{l}}\leq p_{k}\\ 1\Leftrightarrow d_{k_{l}}>p_{k}\; \end{array}\right.,\;l=1,\ldots ,4$$

Correction for level criterion (13):

(13) $$\left\{ \begin{array}{c} P_{k}\left({d_{k}}_{2}\right)=0⇐\frac{q_{k}-{d_{k}}_{1}}{{d_{k}}_{2}-{d_{k}}_{1}}>0.5\wedge {d_{k}}_{1}<q_{k}\leq {d_{k}}_{2}\\ P_{k}\left({d_{k}}_{3}\right)=1⇐\frac{p_{k}-{d_{k}}_{4}}{{d_{k}}_{3}-{d_{k}}_{4}}>0.5\wedge {d_{k}}_{3}\leq p_{k}<{d_{k}}_{4} \end{array}\right.$$

V-shaped criterion with indifference area (14):

(14) $$\tilde{P_{k}}\left(\tilde{d_{k}}\right)=\left(P_{k}\left({d_{k}}_{1}\right),P_{k}\left({d_{k}}_{2}\right),P_{k}\left({d_{k}}_{3}\right),P_{k}\left({d_{k}}_{4}\right)\right)=\left\{ \begin{array}{c} 0\Leftrightarrow d_{k_{l}}\leq q_{k}\\ \frac{d_{k_{l}}-q_{k}}{p_{k}-q_{k}}\Leftrightarrow q_{k}<d_{k_{l}}\leq p_{k}\\ 1\Leftrightarrow d_{k_{l}}>p_{k}\; \end{array}\right.,\;l=1,\ldots ,4$$

Correction for V-shaped criterion with indifference area (15):

(15) $$\left\{ \begin{array}{c} P_{k}\left({d_{k}}_{2}\right)=0⇐\frac{q_{k}-{d_{k}}_{1}}{{d_{k}}_{2}-{d_{k}}_{1}}>0.5\wedge {d_{k}}_{1}<q_{k}\leq {d_{k}}_{2}\\ P_{k}\left({d_{k}}_{3}\right)=1⇐\frac{p_{k}-{d_{k}}_{4}}{{d_{k}}_{3}-{d_{k}}_{4}}>0.5\wedge {d_{k}}_{3}\leq p_{k}<{d_{k}}_{4} \end{array}\right.$$

Gaussian criterion (16):

(16) $$\tilde{P_{k}}\left(\tilde{d_{k}}\right)=\left(P_{k}\left({d_{k}}_{1}\right),P_{k}\left({d_{k}}_{2}\right),P_{k}\left({d_{k}}_{3}\right),P_{k}\left({d_{k}}_{4}\right)\right)=\left\{ \begin{array}{c} 0\Leftrightarrow d_{k_{l}}\leq 0\\ 1-\text{exp}\left(\frac{-{d_{k_{l}}}^{2}}{2{s_{k}}^{2}}\right)\Leftrightarrow d_{k_{l}}>0 \end{array}\right.,\;l=1,\ldots ,4$$

Correction for Gaussian criterion (17):

(17) $$P_{k}\left({d_{k}}_{2}\right)=0⇐\frac{-{d_{k}}_{1}}{{d_{k}}_{2}-{d_{k}}_{1}}>0.5\wedge {d_{k}}_{1}<0\leq {d_{k}}_{2}$$

**Fig. 4:**
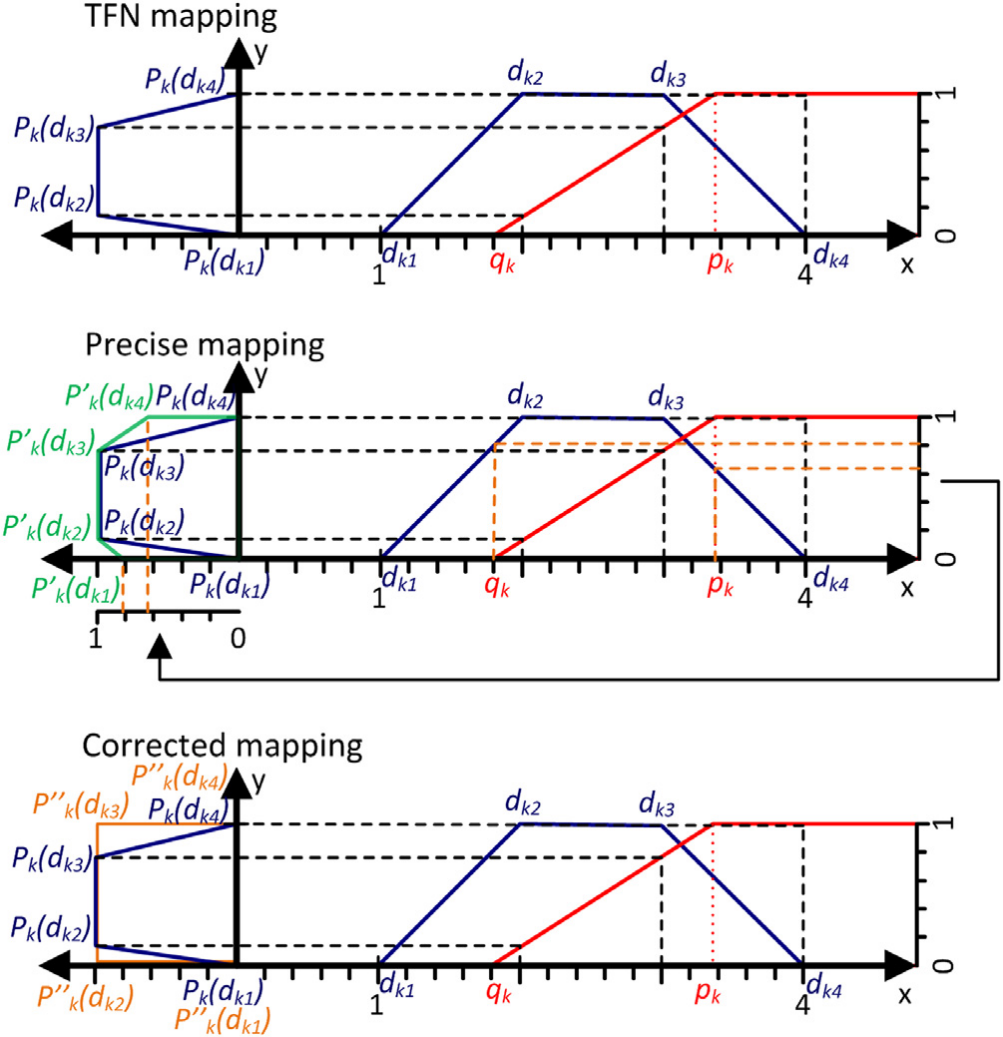
TFN mapping, precise mapping and correction mapping.

Fig. 4 shows an example of an approximation error that occurs during the TFN mapping, the correct mapping result and the operation of the correction mechanism used in the NEAT F-PROMETHEE method.

Fig. 4 contains a TFN $\tilde{d_{k}}=\left({d_{k}}_{1},{d_{k}}_{2},{d_{k}}_{3},{d_{k}}_{4}\right)=\left(1,2,3,4\right)$ mapped with the use of the linear preference function, called V-shaped criterion with indifference area, with parameters $q_{k}=1.8,\;\;p_{k}=3.4$. For TFN mapping, according to the formula (14), the mapping result will be a TFN $\tilde{P_{k}}\left(\tilde{d_{k}}\right)=\left(P_{k}\left({d_{k}}_{1}\right),P_{k}\left({d_{k}}_{2}\right),P_{k}\left({d_{k}}_{3}\right),P_{k}\left({d_{k}}_{4}\right)\right)=\left(0,\frac{2-1.8}{3.4-1.8},\frac{3-1.8}{3.4-1.8},1\right)=\left(0,0.125,0.75,1\right)$. According to the membership function defined for a TFN [16,17], the fuzzy number values at the indicated points are $\mu_{\tilde{P_{k}}\left(\tilde{d_{k}}\right)}\left(y\right)=\left(0,1,1,0\right)$. In the case of precision mapping, since none of the preference functions used is an injection (so the preference function can take the same values for two different values on the *x*-axis), the mapping function described by the formula (18) [18,19] should be used to determine the value of the fuzzy number in points (0,0.125,0.75,1).

(18) $$\mu_{\tilde{P_{k}}\left(\tilde{d_{k}}\right)}\left(y\right)=\max_{y=P_{k}\left(x\right)}\mu_{\tilde{d_{k}}}\left(x\right)$$

Based on the formula (18) and the maximum values $\tilde{d_{k}}$ in points $q_{k}$ and $p_{k}$, which, based on the formula (1), are $\mu_{\tilde{d_{k}}}\left(q_{k}\right)=\left(\frac{q_{k}-{d_{k}}_{1}}{{d_{k}}_{2}-{d_{k}}_{1}}\Leftrightarrow {d_{k}}_{1}<q_{k}\leq {d_{k}}_{2}\right)=\frac{1.8-1}{2-1}=0.8$ and $\mu_{\tilde{d_{k}}}\left(p_{k}\right)=\left(\frac{{d_{k}}_{4}-p_{k}}{{d_{k}}_{4}-{d_{k}}_{3}}\Leftrightarrow {d_{k}}_{3}\leq p_{k}<{d_{k}}_{4}\right)=\frac{4-3.4}{4-3}=0.6$ it, is easy to see that the fuzzy number values in points (0,0.125,0.75,1) should be $\mu_{\tilde{P_{k}}\left(\tilde{d_{k}}\right)}\left(y\right)=\left(0.8,1,1,0.6\right)$. Therefore, TNF mapping generates a relatively large approximation error. That is why, in the NEAT F-PROMETHEE method, a shape correction of the obtained TFN was introduced to make it as close as possible to the result of precise mapping. The code of the MATLAB function, which calculates fuzzy deviation and the values of the correction preference function, is shown below.







A separate MATLAB function contains a procedure to check if a correction is required. From the formulae 6–(17), it can be seen that the conditions of correction for all preference functions are similar and can be recorded as the formula (19):

(19) $$\left\{ \begin{array}{c} P_{k}\left({d_{k}}_{2}\right)=0⇐\frac{t-{d_{k}}_{1}}{{d_{k}}_{2}-{d_{k}}_{1}}>0.5\wedge {d_{k}}_{1}<t\leq {d_{k}}_{2}\\ P_{k}\left({d_{k}}_{3}\right)=1⇐\frac{u-{d_{k}}_{4}}{{d_{k}}_{3}-{d_{k}}_{4}}>0.5\wedge {d_{k}}_{3}\leq u<{d_{k}}_{4} \end{array}\right.$$

However, the *t* and *u* variables allow distinguishing indifference, weak preference and strict preference relationships. Depending on the preference function used, *t* and *u* take different values:

• for the usual criterion: t=0, u=0,
• for the U-shaped criterion: $t=q_{k}$, $u=q_{k}$,
• for the V-shaped criterion: t=0, $u=p_{k}$,
• for the level criterion and V-shaped criterion with indifference area: $t=q_{k}$, $u=p_{k}$,
• for the Gaussian criterion: t=0, u=∞.

In the case of u=∞ for the Gaussian criterion, it should be clarified that this value is due to the property of the Gaussian preference function, which asymptotically tends to 1, and therefore does not allow obtaining strict preference [20]. In the case of u=∞there is an obvious contradiction because for a correction to occur, the value ${d_{k}}_{4}$ would have to be greater than infinity. Therefore, this correction function is not used for the Gaussian criterion. The code of the MATLAB function for checking the conditions of correction is as follows.



After the mapping and correction process, the weights of the criteria $\tilde{wf_{j}}=\left(wf_{1},wf_{2},wf_{3},wf_{4}\right)$ are defuzzified and normalised. As a result, a new vector of weights of criteria $W=\{w_{1},w_{2},\ldots ,w_{n}\}$ is obtained. These actions are necessary to keep the scale [−1,1] for the obtained solution, as it is done in the classic crisp PROMETHEE method. The defuzzification is performed using the Centroid method, described by the formula (20):

(20) $$w_{j}=\frac{wf_{j_{3}}^{2}+wf_{j_{4}}^{2}+wf_{j_{3}}fw_{j_{4}}-wf_{j_{1}}^{2}-wf_{j_{2}}^{2}-wf_{j_{1}}wf_{j_{2}}}{3\left(wf_{j_{3}}+wf_{j_{4}}-wf_{j_{1}}-wf_{j_{2}}\right)}$$

The Centroid method, unlike the Bisector method, does not allow defuzzying crisp numbers (where $w_{j_{1}}=w_{j_{2}}=w_{j_{3}}=w_{j_{4}}$), so for such numbers a simple assignment$\;w_{j}=w_{j_{1}}$ should be used instead of the formula (20). The purpose of the normalisation is to bring the sum of all weights to 1 ($\sum_{j=1}^{n}w_{j}=1$) and to define proportionally the weights of each criterion. It is performed according to the formula (21):

(21) $$w_{j}=\frac{w_{j}}{\sum_{i=1}^{n}w_{i}}$$

The defuzzification and normalisation has been implemented in the appropriate MATLAB function.



In the next step, preferences are aggregated between the different pairs of decision alternatives and fuzzy outranking flows are calculated for each alternative. The aggregation of preferences is done according to the formula (22):

(22) $$\Lambda_{\tilde{a_{i}},\tilde{a_{j}}\in \tilde{A}}\wedge_{w_{k}\in W}\tilde{\pi }\left(\tilde{a_{i}},\tilde{a_{j}}\right)=\sum_{k=1}^{n}\tilde{P_{k}}\left(\tilde{d_{k}}\left(\tilde{a_{i}},\tilde{a_{j}}\right)\right)\times w_{k}$$

After the aggregation of preferences, fuzzy outranking flows are calculated 23–(25):

(23) $$\Lambda_{\tilde{a_{i}}\in \tilde{A}}\tilde{\phi^{+}}\left(\tilde{a_{i}}\right)=\frac{\sum_{j=1,\;j\neq i}^{m}\tilde{\pi }\left(\tilde{a_{i}},\tilde{a_{j}}\right)}{m-1}$$

(24) $$\Lambda_{\tilde{a_{i}}\in \tilde{A}}\tilde{\phi^{-}}\left(\tilde{a_{i}}\right)=\frac{\sum_{j=1,\;j\neq i}^{m}\tilde{\pi }\left(\tilde{a_{j}},\tilde{a_{i}}\right)}{m-1}$$

(25) $$\Lambda_{\tilde{a_{i}}\in \tilde{A}}\tilde{\phi_{net}}\left(\tilde{a_{i}}\right)=\tilde{\phi^{+}}\left(\tilde{a_{i}}\right)⊖\tilde{\phi^{-}}\left(\tilde{a_{i}}\right)$$

The given operations have been implemented as a function in MATLAB environment.





The obtained values of fuzzy outranking flows are then defuzzified using the Centroid method 26–(28), similar to the fuzzy weights:

(26) $$\Lambda_{\tilde{a_{i}}\in \tilde{A}}\phi^{+}\left(\tilde{a_{i}}\right)=\frac{\phi^{+}{\left(\tilde{a_{i}}\right)}_{3}^{2}+\phi^{+}{\left(\tilde{a_{i}}\right)}_{4}^{2}+\phi^{+}{\left(\tilde{a_{i}}\right)}_{3}\phi^{+}{\left(\tilde{a_{i}}\right)}_{4}-\phi^{+}{\left(\tilde{a_{i}}\right)}_{1}^{2}-\phi^{+}{\left(\tilde{a_{i}}\right)}_{2}^{2}-\phi^{+}{\left(\tilde{a_{i}}\right)}_{1}\phi^{+}{\left(\tilde{a_{i}}\right)}_{2}}{3\left(\phi^{+}{\left(\tilde{a_{i}}\right)}_{3}+\phi^{+}{\left(\tilde{a_{i}}\right)}_{4}-\phi^{+}{\left(\tilde{a_{i}}\right)}_{1}-\phi^{+}{\left(\tilde{a_{i}}\right)}_{2}\right)}$$

(27) $$\Lambda_{\tilde{a_{i}}\in \tilde{A}}\phi^{-}\left(\tilde{a_{i}}\right)=\frac{\phi^{-}{\left(\tilde{a_{i}}\right)}_{3}^{2}+\phi^{-}{\left(\tilde{a_{i}}\right)}_{4}^{2}+\phi^{-}{\left(\tilde{a_{i}}\right)}_{3}\phi^{-}{\left(\tilde{a_{i}}\right)}_{4}-\phi^{-}{\left(\tilde{a_{i}}\right)}_{1}^{2}-\phi^{-}{\left(\tilde{a_{i}}\right)}_{2}^{2}-\phi^{-}{\left(\tilde{a_{i}}\right)}_{1}\phi^{-}{\left(\tilde{a_{i}}\right)}_{2}}{3\left(\phi^{-}{\left(\tilde{a_{i}}\right)}_{3}+\phi^{-}{\left(\tilde{a_{i}}\right)}_{4}-\phi^{-}{\left(\tilde{a_{i}}\right)}_{1}-\phi^{-}{\left(\tilde{a_{i}}\right)}_{2}\right)}$$

(28) $$\Lambda_{\tilde{a_{i}}\in \tilde{A}}\phi_{net}\left(\tilde{a_{i}}\right)=\frac{\phi_{net}{\left(\tilde{a_{i}}\right)}_{3}^{2}+\phi_{net}{\left(\tilde{a_{i}}\right)}_{4}^{2}+\phi_{net}{\left(\tilde{a_{i}}\right)}_{3}\phi_{net}{\left(\tilde{a_{i}}\right)}_{4}-\phi_{net}{\left(\tilde{a_{i}}\right)}_{1}^{2}-\phi_{net}{\left(\tilde{a_{i}}\right)}_{2}^{2}-\phi_{net}{\left(\tilde{a_{i}}\right)}_{1}\phi_{net}{\left(\tilde{a_{i}}\right)}_{2}}{3\left(\phi_{net}{\left(\tilde{a_{i}}\right)}_{3}+\phi_{net}{\left(\tilde{a_{i}}\right)}_{4}-\phi_{net}{\left(\tilde{a_{i}}\right)}_{1}-\phi_{net}{\left(\tilde{a_{i}}\right)}_{2}\right)}$$

Similarly to the defuzzification of weights of criteria, if the outranking flows are crisp numbers (e.g.$\;\phi^{+}{\left(\tilde{a_{i}}\right)}_{1}=\phi^{+}{\left(\tilde{a_{i}}\right)}_{2}=\phi^{+}{\left(\tilde{a_{i}}\right)}_{3}=\phi^{+}{\left(\tilde{a_{i}}\right)}_{4}$), one should use a simple assignment (e.g. $\phi^{+}\left(\tilde{a_{i}}\right)=\phi^{+}{\left(\tilde{a_{i}}\right)}_{1}$). The MATLAB code responsible for defuzzification of outranking flows is shown below.



### Generating rankings and displaying the results of the method

On the basis of the defuzzified values $\phi_{net},$ a full NEAT F-PROMETHEE II (total order) ranking is generated, while the values $\phi^{+}$ i $\phi^{-}$ are the basis for constructing the rankings that are later used in the NEAT F-PROMETHEE I (partial order) ranking. The MATLAB function responsible for this assigns each alternative an appropriate rank in the full ranking and the rankings $\phi^{+}$ i $\phi^{-}$.



After three rankings have been constructed, they are presented to the decision maker using the *showResults* function together with the defuzzifieded values of outranking flows ($\phi_{net}$, $\phi^{+}$, $\phi^{-}$), which are the basis for building these rankings.



In addition to presenting the rankings in the form of numerical values, in the NEAT F-PROMETHEE implementation, the results are also presented in a graphic form. This is performed by *plotResults* and *plotPartialOrder* functions. The *plotResults* function presents graphs $\phi_{net}$, $\phi^{+}$ i $\phi^{-}$ containing fuzzy and crisp outranking flows for individual alternatives, together with the positions of these alternatives in the rankings. The chart $\phi^{+}$ shows how much a given alternative is outranking the others, while the chart $\phi^{-}$ depicts how much a given alternative is outranked by the others. In turn, the graph $\phi_{net}$ illustrates the total order of alternatives, in other words, it presents a solution to a decision-making problem using the NEAT F-PROMETHEE II method. It should be added that there may be two preference relationships in the total order of alternatives: (1) indifference between $\tilde{a_{i}}$ and $\tilde{a_{j}}$ ($\tilde{a_{i}}\;I\;\tilde{a_{j}}$) when $\phi_{net}\left(\tilde{a_{i}}\right)=\phi_{net}\left(\tilde{a_{j}}\right)$, (2) preference of $\tilde{a_{i}}$ over $\tilde{a_{j}}$ ($\tilde{a_{i}}≻\tilde{a_{j}}$) when $\phi_{net}\left(\tilde{a_{i}}\right)>\phi_{net}\left(\tilde{a_{j}}\right)$.





The *plotPartialOrder* function presents in a graphical form a partial order of alternatives, constructed on the basis of $\phi^{+}$ and $\phi^{-}$ rankings. There may be three preference relationships in the partial order of alternatives: (1) indifference between $\tilde{a_{i}}$ and $\tilde{a_{j}}$ ($\tilde{a_{i}}\;I\;\tilde{a_{j}}$) when $\left(\tilde{\phi^{+}}\left(\tilde{a_{i}}\right)=\tilde{\phi^{+}}\left(\tilde{a_{j}}\right)\right)\;\wedge \;\left(\tilde{\phi^{-}}\left(\tilde{a_{i}}\right)=\tilde{\phi^{-}}\left(\tilde{a_{j}}\right)\right)$, (2) preference of $\tilde{a_{i}}$ over $\tilde{a_{j}}$ ($\tilde{a_{i}}≻\tilde{a_{j}}$) when $\left(\tilde{\phi^{+}}\left(\tilde{a_{i}}\right)\geq \tilde{\phi^{+}}\left(\tilde{a_{j}}\right)\right)\;\wedge \;\left(\tilde{\phi^{-}}\left(\tilde{a_{i}}\right)\leq \tilde{\phi^{-}}\left(\tilde{a_{j}}\right)\right)$, where one of the inequalities is strict, (3) incomparability between $\tilde{a_{i}}$ over $\tilde{a_{j}}$ ($\tilde{a_{i}}\;R\;\tilde{a_{j}}$) when $\left(\tilde{\phi^{+}}\left(\tilde{a_{i}}\right)>\tilde{\phi^{+}}\left(\tilde{a_{j}}\right)\;\wedge \;\tilde{\phi^{-}}\left(\tilde{a_{i}}\right)>\tilde{\phi^{-}}\left(\tilde{a_{j}}\right)\right)\vee \left(\tilde{\phi^{+}}\left(\tilde{a_{i}}\right)<\tilde{\phi^{+}}\left(\tilde{a_{j}}\right)\;\wedge \;\tilde{\phi^{-}}\left(\tilde{a_{i}}\right)<\tilde{\phi^{-}}\left(\tilde{a_{j}}\right)\right)$. The partial order presents an order of alternatives using the indicated preference relationships. It should be noted that the graphic presentation of the partial order shows indifference and preference relations in the form of edges connecting the alternatives directly or indirectly, while incomparability is represented by the lack of direct or indirect connection.





In the developed implementation of the NEAT F-PROMETHEE method, apart from the proprietary functions, the *distinguishable_colors* [21] and *line2arrow* [22] functions were also used.

### Method validation

The correctness of the implementation of the NEAT F-PROMETHEE method has been verified by solving the decision problem on the basis of selecting a "green" supplier of electronic items for a manufacturing company in order to reduce costs at the manufacturing stage of finished products. In the decision-making process, 4 suppliers $\tilde{A}=\left\{\tilde{a_{1}},\tilde{a_{2}},\tilde{a_{3}},\tilde{a_{4}}\right\}$, have been considered, assessing them against 6 criteria $C=\{c_{1},c_{2},c_{3},c_{4},c_{5},c_{6}\}$. Table 1 shows the parameters of the different decision alternatives and Table 2 includes the preference model used, i.e. the weights of the criteria, preference directions, preference functions and thresholds.

**Table 1:** Fuzzy assessments of alternatives.

Criterion	$\tilde{a_{1}}$	$\tilde{a_{2}}$	$\tilde{a_{3}}$	$\tilde{a_{4}}$
c_1_ – Price [thousand PLN]	130	117	96	136
c_2_ – Quality [Linguistic]	G	MG	G	F
c_3_ – Green product [Linguistic]	G	MG	MP	MG
c_4_ – Lead time [Days]	(28,30,30,32)	(24,26,30,30)	(20,22,24,26)	(22,22,24,24)
c_5_ – Reliability [Linguistic]	G	G	MG	VG
c_6_ – Green delivery [Linguistic]	P	G	VP	MP

**Table 2:** Preference model.

Criterion	Weight	Preference direction	Preference function	Indifference threshold (*q*)	Preference threshold (*p*)	Gaussian threshold (*s*)
c_1_ – Price [thousand PLN]	H	Min	V-shape with indifference	5	40	–
c_2_ – Quality [Linguistic]	H	Max	Usual	–	–	–
c_3_ – Green product [Linguistic]	M	Max	Level	0	1	–
c_4_ – Lead time [Days]	MH	Min	V-shape	–	5	–
c_5_ – Reliability [Linguistic]	VH	Max	V-shape with indifference	0.5	1.5	–
c_6_ – Green delivery [Linguistic]	ML	Max	Gaussian	–	–	1

**Fig. 5:**
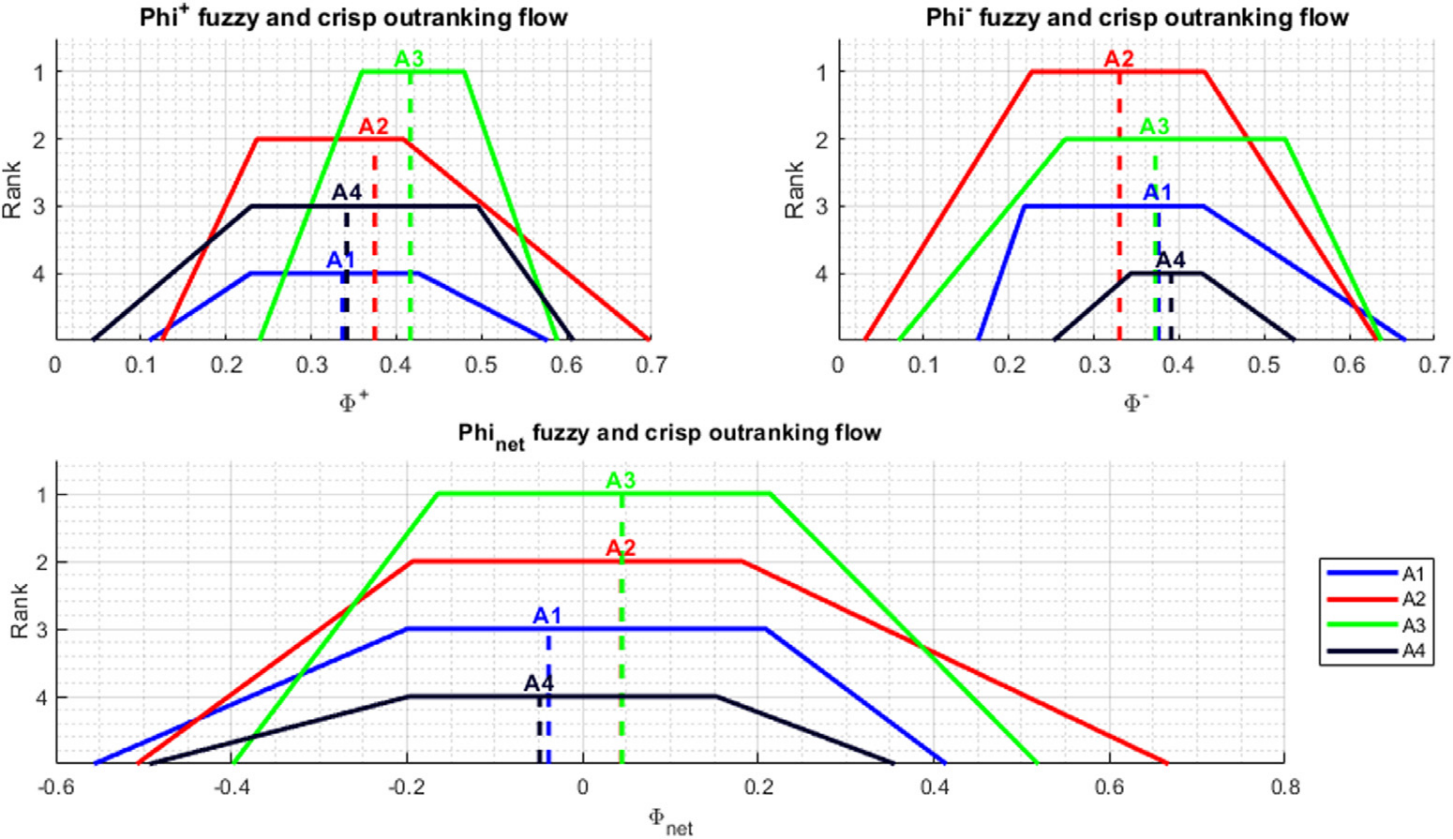
Graphical representation of outranking flows ($\phi_{net}$, $\phi^{+}$, $\phi^{-}$) and corresponding rankings (NEAT F-PROMETHEE II).

**Fig. 6:**
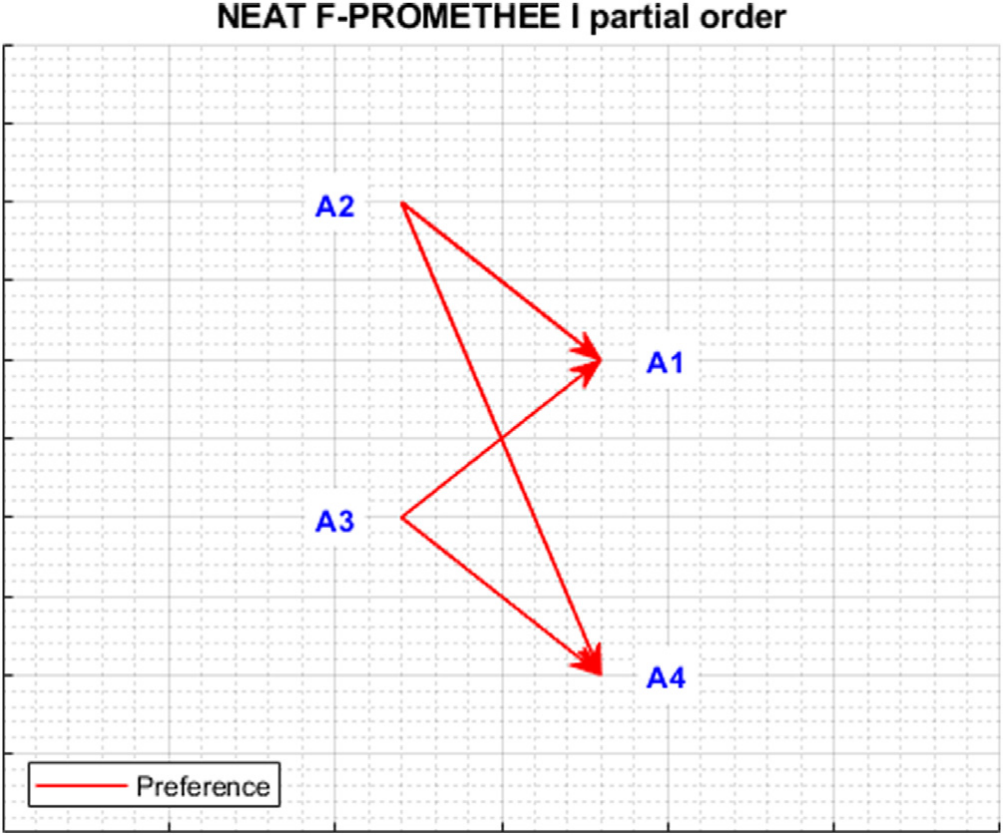
Partial order NEAT F-PROMETHEE I.

The application of the NEAT F-PROMETHEE method presented in the article has made it possible to obtain a solution to the decision problem, presented in Table 3 and Figs. 5 and 6.

**Table 3:** The values of outranking flows and rankings of alternatives (NEAT F-PROMETHEE).

$\tilde{A}$	$\phi^{+}$	Rank $\phi^{+}$	$\phi^{-}$	Rank $\phi^{-}$	$\phi_{net}$	Rank NEAT F-PROMETHEE II ($\phi_{net}$)	Rank NEAT F-PROMETHEE I ($\phi^{+}\circ \phi^{-}$)
$\tilde{a_{1}}$	0.3368	4	0.3754	3	−0.0386	3	$\tilde{a_{2}}≻\tilde{a_{1}};\;\tilde{a_{3}}≻\tilde{a_{1}}$
$\tilde{a_{2}}$	0.3747	2	0.3301	1	0.0443	2	$\tilde{a_{2}}≻\tilde{a_{1}};\;\tilde{a_{2}}≻\tilde{a_{4}}$
$\tilde{a_{3}}$	0.4165	1	0.3721	2	0.0448	1	$\tilde{a_{3}}≻\tilde{a_{1}};\;\tilde{a_{3}}≻\tilde{a_{4}}$
$\tilde{a_{4}}$	0.3419	3	0.3906	4	−0.0489	4	$\tilde{a_{2}}≻\tilde{a_{4}};\;\tilde{a_{3}}≻\tilde{a_{4}}$

Figs. 5 and 6, generated using *plotResults.m* and *plotPartialOrder.m* functions, enable an analysis of the obtained solution. Fig. 5 shows the fuzzy and disinfected values of alternatives, as well as the order of alternatives, separately for $\phi^{+}$, $\phi^{-}$ and $\phi_{net}$ rankings. The ranking $\phi^{+}$ allows us to conclude that the alternative, which is outranking all others the most, is $\tilde{a_{3}}$. It should be noted that the support of fuzzy numbers indicates that $\tilde{a_{3}}$ can be outranked in the ranking $\phi^{+}$ by an alternative $\tilde{a_{2}}$, or even $\tilde{a_{4}}$. In turn, according to the ranking $\phi^{-},$ the alternative most outranked by the others is$\;\tilde{a_{4}}$, although the analysis of fuzzy numbers indicates the possibility that the other alternatives will be outranked by $\tilde{a_{4}}$. Finally, when analysing the ranking $\phi_{net}$ the solution to the decision problem is the following total order of alternatives: $\tilde{a_{3}}≻\tilde{a_{2}}≻\tilde{a_{1}}≻\tilde{a_{4}}$. However, the total order of alternatives obtained is characterised by a relatively high degree of uncertainty, as evidenced by the wide range of kernels and support for the fuzzy numbers obtained. As regards Fig. 6, which shows the partial order of the alternatives, it should be concluded that the dominant alternatives are $\tilde{a_{3}}$ and $\tilde{a_{2}}$, which are preferred over $\tilde{a_{1}}$ and $\tilde{a_{4}}$. This calculation example shows the usefulness of the fuzzy approach to interpret the degree of uncertainty of the solution obtained.

**Fig. 7:**
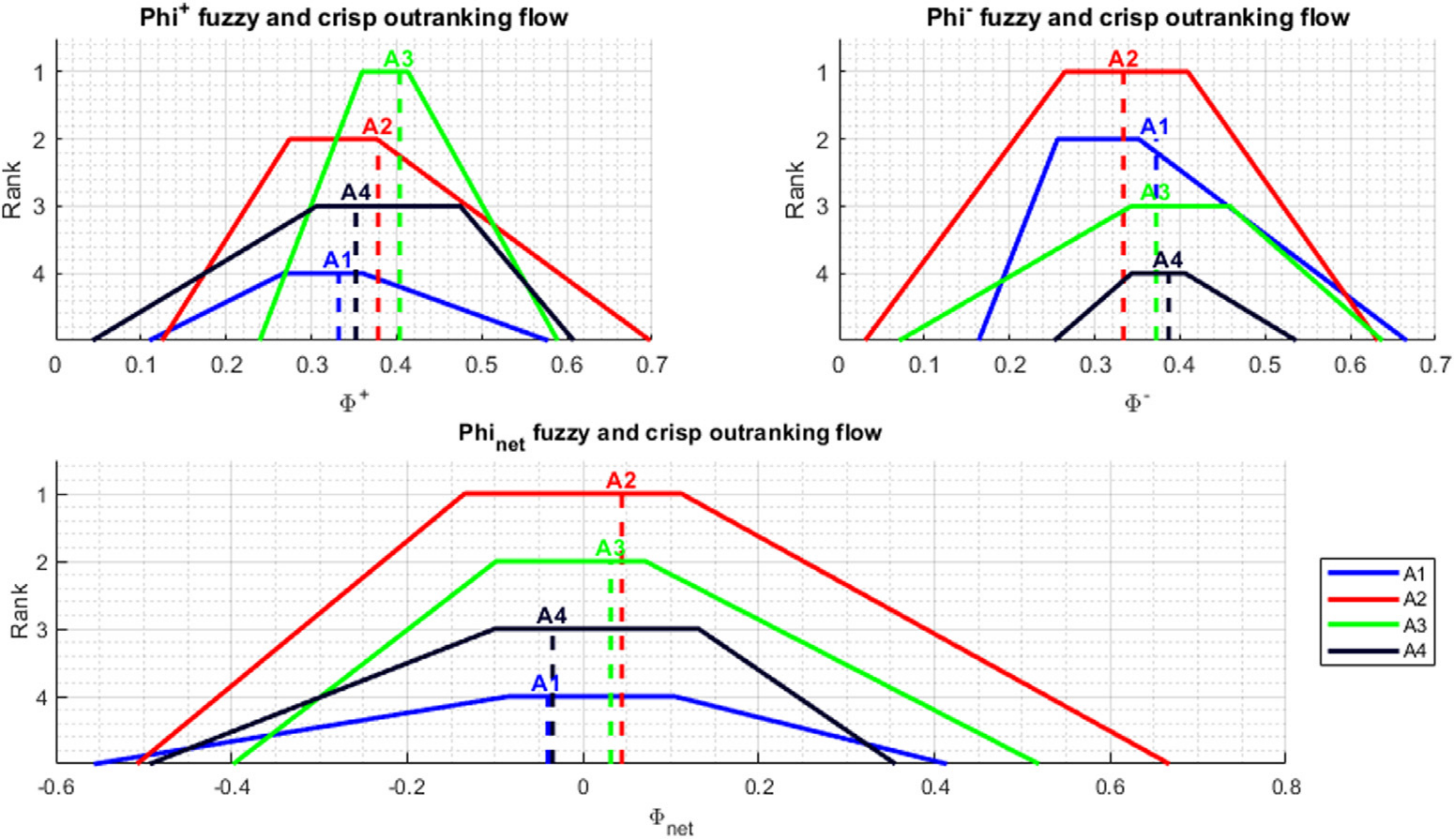
Graphical representation of outranking flows ($\phi_{net}$, $\phi^{+}$, $\phi^{-}$) and their corresponding rankings obtained without mapping correction (Fuzzy PROMETHEE II).

**Fig. 8:**
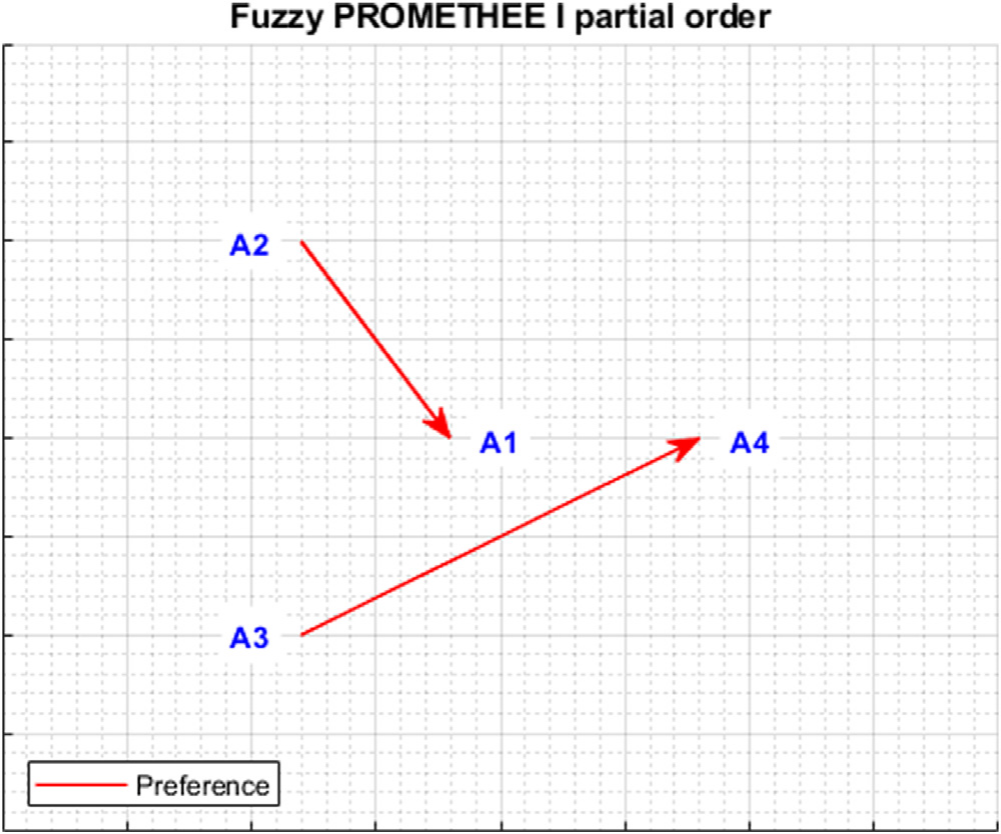
Partial order obtained without mapping correction (Fuzzy PROMETHEE I).

Apart from the verification of the correctness of the implementation of the NEAT F-PROMETHEE method in the MATLAB environment, the operation of the correction of mapping errors (see Formulae (6)-(19)) and the impact of the correction on the obtained solution were also verified. For this purpose, the presented decision problem was solved using fuzzy PROMETHEE without correction. The solution obtained in this way is shown in Table 4 and Figs. 7 and 8.

**Table 4:** The values of outranking flows and the rankings of alternatives obtained without mapping correction (Fuzzy PROMETHEE).

$\tilde{A}$	$\phi^{+}$	Rank $\phi^{+}$	$\phi^{-}$	Rank $\phi^{-}$	$\phi_{net}$	Rank Fuzzy PROMETHEE II ($\phi_{net}$)	Rank Fuzzy PROMETHEE I ($\phi^{+}\circ \phi^{-}$)
$\tilde{a_{1}}$	0.3320	4	0.37207	3	−0.0399	4	$\tilde{a_{2}}≻\tilde{a_{1}}$
$\tilde{a_{2}}$	0.3784	2	0.3336	1	0.0441	1	$\tilde{a_{2}}≻\tilde{a_{1}}$
$\tilde{a_{3}}$	0.4038	1	0.37209	2	0.0317	2	$\tilde{a_{3}}≻\tilde{a_{4}}$
$\tilde{a_{4}}$	0.3522	3	0.3867	4	−0.0346	3	$\tilde{a_{3}}≻\tilde{a_{4}}$

**Fig. 9:**
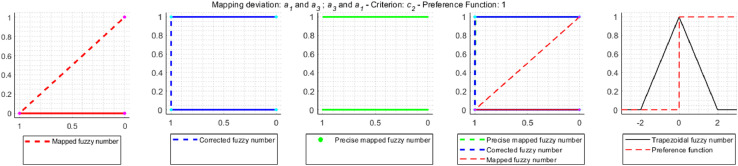
Error and correction during the mapping of deviations $\tilde{d_{2}}\left(\tilde{a_{1}},\tilde{a_{3}}\right)$ and $\tilde{d_{2}}\left(\tilde{a_{3}},\tilde{a_{1}}\right).$

**Fig. 10:**
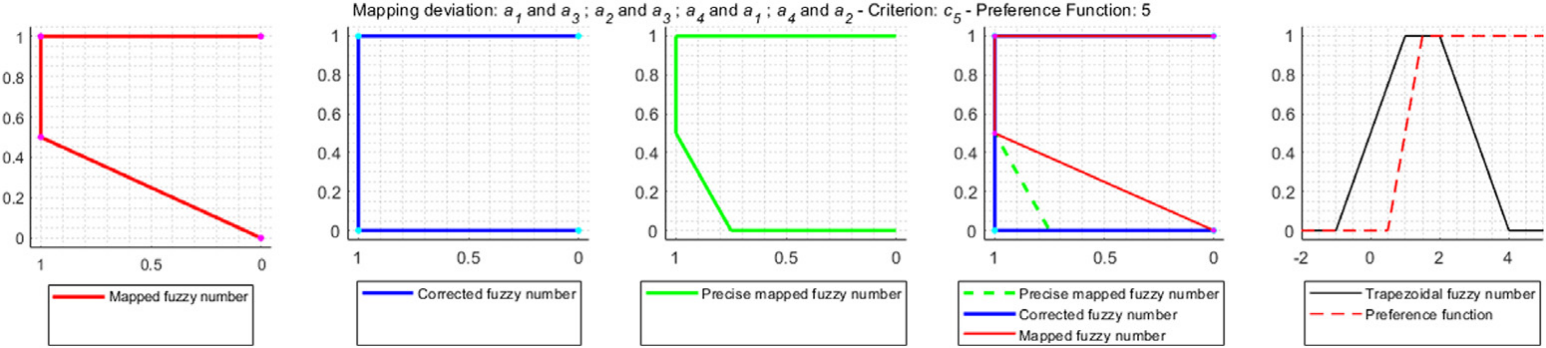
Error and correction during the mapping of deviations $\tilde{d_{5}}\left(\tilde{a_{1}},\tilde{a_{3}}\right)$, $\tilde{d_{5}}\left(\tilde{a_{2}},\tilde{a_{3}}\right)$, $\tilde{d_{5}}\left(\tilde{a_{4}},\tilde{a_{1}}\right)$ and $\tilde{d_{5}}\left(\tilde{a_{4}},\tilde{a_{2}}\right).$

**Fig. 11:**
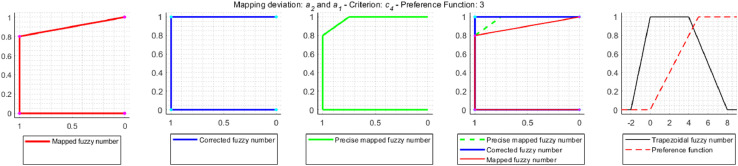
Error and correction during the mapping of deviations $\tilde{d_{4}}\left(\tilde{a_{2}},\tilde{a_{1}}\right).$

**Fig. 12:**
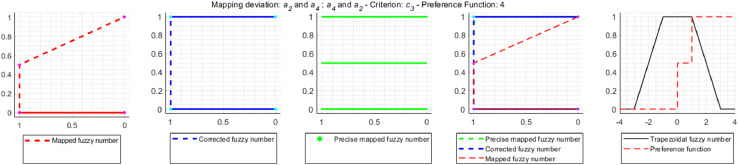
Error and correction during the mapping of deviations $\tilde{d_{3}}\left(\tilde{a_{2}},\tilde{a_{4}}\right)$ and $\tilde{d_{3}}\left(\tilde{a_{4}},\tilde{a_{2}}\right).$

A comparison of Table 3 and Figs. 4 and 5 (solution with correction) with Table 4 and Figs. 6 and 7 (solution without correction) shows that, in the absence of correction, there was a change in the ranking$\;\phi_{net}$ in positions 1–2 and 3–4. In addition, the partial order of alternatives, namely preference relationships$\;\tilde{a_{3}}≻\tilde{a_{1}}$**,** $\tilde{a_{2}}≻\tilde{a_{4}}$ were converted into incomparability relationships $\tilde{a_{1}\;}R\;\tilde{a_{3}}$**,** $\tilde{a_{2}}\;R\;\tilde{a_{4}}$. In order to clearly determine which solution is correct, approximation errors resulting from the use of trapezoidal fuzzy numbers instead of accurate fuzzy mapping were examined. As a result of the study, it was found that in the decision problem under consideration, the approximation error occurs relatively often, because in 32 cases out of 72 mappings performed, i.e. in 44% of cases. On the other hand, the correction is made in 9 mappings, i.e. 12.5% of all cases and 28% of the mappings are affected by an error. The mappings for which the correction is made are shown in Figs. 9–12.

In addition to the mapping analysis during which correction is applied, cumulative mapping errors were also examined, with and without correction. Mapping errors were calculated for each of the preference functions, by defuzzyfing fuzzy numbers obtained using precise mapping (29) and trapezoidal fuzzy numbers (30) obtained using non-corrected and corrected mapping. Then the errors for each criterion were summed up, separately for each preference function *P*, where *P∈ {usual criterion, V-shaped criterion, level criterion, V-shaped criterion with indifference area, Gaussian criterion}* (31) (the U-shaped criterion function was not applied in the decision-making model under consideration).

(29) $$Dc\left(\mu_{\tilde{P_{k}}\left(\tilde{d_{k}}\right)}\right)=\frac{\int_{y_{min}}^{y_{max}}y\cdot \mu_{\tilde{P_{k}}\left(\tilde{d_{k}}\right)}\left(y\right)dy}{\int_{y_{min}}^{y_{max}}\mu_{\tilde{P_{k}}\left(\tilde{d_{k}}\right)}\left(y\right)dy}$$

(30) $$Dt\left(\mu_{\tilde{P_{k}}\left(\tilde{d_{k}}\right)}\right)=\frac{P_{k}{\left({d_{k}}_{3}\right)}^{2}+P_{k}{\left({d_{k}}_{4}\right)}^{2}+P_{k}\left({d_{k}}_{3}\right)P_{k}\left({d_{k}}_{4}\right)-P_{k}{\left({d_{k}}_{1}\right)}^{2}-P_{k}{\left({d_{k}}_{2}\right)}^{2}-P_{k}\left({d_{k}}_{1}\right)P_{k}\left({d_{k}}_{2}\right)}{3\left(P_{k}\left({d_{k}}_{3}\right)+P_{k}\left({d_{k}}_{4}\right)-P_{k}\left({d_{k}}_{1}\right)-P_{k}\left({d_{k}}_{2}\right)\right)}$$

(31) $$error\left(P\right)=\sum_{k=1}^{n}\left|Dc\left(\mu_{\tilde{P_{k}}\left(\tilde{d_{k}}\right)}\right)-Dt\left(\mu_{\tilde{P_{k}}\left(\tilde{d_{k}}\right)}\right)\right|$$

The error values obtained during mapping with and without correction are shown in Table 5.

**Table 5:** Mapping errors with and without correction.

Preference function	Error with correction	Error without correction	Improvement of the results [%]
Usual criterion	0.9956	1.3272	25%
V-shaped criterion	0.062	0.0879	29%
Level criterion	0.3864	0.607	36%
V-shaped criterion with indifference area	0.3388	0.6056	44%
Gaussian criterion	0.1467	0.1467	0%

**Fig. 13:**
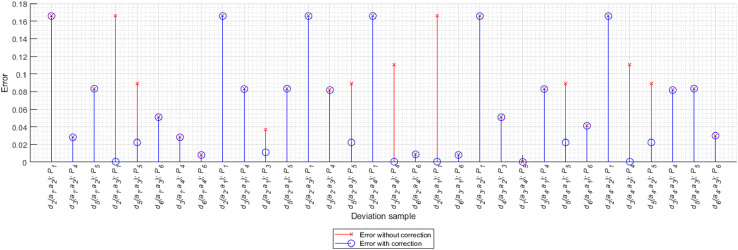
Diagram of errors during mapping.

The results presented in Table 5 clearly show that the solution obtained by applying the correction is less error prone. This is confirmed by the diagram of errors during mapping, shown in Fig. 13. The analysis of Fig. 13 indicates that in the decision problem under consideration, if a correction is made, it reduces the mapping error in each case.

The presented analyses allow us to conclude that the decision problem solution obtained using the NEAT F-PROMETHEE method (with correction) has a smaller error than the solution obtained using Fuzzy PROMETHEE (without correction). Therefore, it can be concluded that the ranking of NEAT F-PROMETHEE II ($\tilde{a_{3}}≻\tilde{a_{2}}≻\tilde{a_{1}}≻\tilde{a_{4}}$) and the partial order shown in Fig. 6 is correct. Additionally, the presented calculation example shows that the correction made even when mapping a small number of deviations can significantly change the ranking of considered alternatives.

## Conclusion

The article presents the methodological basis of the NEAT F-PROMETHEE method and the details of its implementation in MATLAB. Moreover, the calculation results of the NEAT F-PROMETHEE method were compared with the standard Fuzzy PROMETHEE method based on TFNs. This comparison was made using the management decision-making problem, which was solved using both multi-criteria decision support methods. The results of the conducted research indicate that the NEAT F-PROMETHEE method allows to obtain more precise results, with a lower error resulting from the use of TFNs. Development of the NEAT F-PROMETHEE implementation in the MATLAB environment will increase the ease of use of this method. This will allow users to focus on better modelling of the decision problems under consideration, instead of worrying about the details related to the correct implementation of the method. As for further directions of research on the NEAT F-PROMETHEE method, in the context of sustainable management, it seems interesting to combine this method with the PROSA method [11,12]. This would allow uncertainty and imprecision to be taken into account in the decision-making problems of sustainable development, where the balance between economic, social and environmental factors is important. Yet another interesting research challenge is the development of GAIA (Geometrical Analysis for Interactive Assistance) [23] for NEAT F-PROMETHEE using TFNs. It would allow analysing the fuzzy decision problem from a descriptive perspective.

## Declaration of Competing Interest

The authors declare that they have no known competing financial interests or personal relationships that could have appeared to influence the work reported in this paper.

## References

[1] Ziemba P. (2021). Multi-criteria approach to stochastic and fuzzy uncertainty in the selection of electric vehicles with high social acceptance, expert systems with applications. Expert Syst. Appl..

[2] Brans J.P., De Smet Y., Greco S., Ehrgott M., Figueira J.R. (2016). PROMETHEE methods. Multiple Criteria Decision Analysis: State of the Art Surveys.

[3] Geldermann J., Spengler T., Rentz O. (2000). Fuzzy outranking for environmental assessment. Case study: iron and steel making industry. Fuzzy Sets Syst..

[4] Drucker P.F. (2010). The Practice of Management.

[5] Puseljic M., Skledar A., Pokupec I. (2015). Decision-making as a management function. Interdiscip. Manag. Res..

[6] Taghavifard M., Khalili-Damghani K., Tavakkoli-Moghaddam R. (2009). Decision making under uncertain and risky situations. Proceedings of the Enterprise Risk Management Symposium.

[7] Chen S.J., Hwang C.L., Chen S.J., Hwang C.L. (1992). Fuzzy sets and their operations. Fuzzy Multiple Attribute Decision Making: Methods and Applications.

[8] Mardani A., Jusoh A., Zavadskas E.K. (2015). Fuzzy multiple criteria decision-making techniques and applications – two decades review from 1994 to 2014. Expert Syst. Appl..

[9] Behzadian M., Kazemzadeh R.B., Albadvi A., Aghdasi M. (2010). PROMETHEE: a comprehensive literature review on methodologies and applications. Eur. J. Oper. Res..

[10] Ziemba P. (2018). NEAT F-PROMETHEE – a new fuzzy multiple criteria decision making method based on the adjustment of mapping trapezoidal fuzzy numbers. Expert Syst. Appl..

[11] Ziemba P. (2020). Multi-criteria stochastic selection of electric vehicles for the sustainable development of local government and state administration units in Poland. Energies.

[12] Ziemba P. (2019). Towards strong sustainability management—a generalized PROSA method. Sustainability..

[13] Ziemba P. (2019). Inter-criteria dependencies-based decision support in the sustainable wind energy management. Energies.

[14] Ziemba P., Becker J. (2019). Analysis of the digital divide using fuzzy forecasting. Symmetry.

[15] Kannchen M., Ziemba P., Borawski M. (2019). Use of the PVM method computed in vector space of increments in decision aiding related to urban development. Symmetry.

[16] Yalçın N., Yapıcı Pehlivan N. (2019). Application of the fuzzy CODAS method based on fuzzy envelopes for hesitant fuzzy linguistic term sets: a case study on a personnel selection problem. Symmetry.

[17] Ziemba P. (2021). Multi-criteria fuzzy evaluation of the planned offshore wind farm investments in Poland. Energies.

[18] Zadeh L.A. (1965). Fuzzy sets. Inf. Control.

[19] Perfilieva I. (2011). Fuzzy Function: Theoretical and Practical Point of View.

[20] van Huylenbroeck G. (1995). The conflict analysis method: bridging the gap between ELECTRE, PROMETHEE and ORESTE. Eur. J. Oper. Res..

[21] Holy T. (2019). Generate Maximally Perceptually-Distinct Colors. https://www.mathworks.com/matlabcentral/fileexchange/29702-generate-maximally-perceptually-distinct-colors.

[22] K. Kearney, line2arrow.m, GitHub. (2019). https://www.github.com/kakearney/line2arrow-pkg (accessed December 17, 2019).

[23] Mareschal B., Brans J.P. (1988). Geometrical representations for MCDA. Eur. J. Oper. Res..

